# Time-Series Analysis of Tumorigenesis in a Murine Skin Carcinogenesis Model

**DOI:** 10.1038/s41598-018-31349-x

**Published:** 2018-08-29

**Authors:** Yoshimasa Aoto, Kazuhiro Okumura, Tsuyoshi Hachiya, Sumitaka Hase, Yuichi Wakabayashi, Fuyuki Ishikawa, Yasubumi Sakakibara

**Affiliations:** 1 Department of Biosciences and Informatics, Keio University, 3-13-1 Hiyoshi, Kohoku-ku, Yokohama, 223-8522 Japan; 2 Department of Carcinogenesis Research, Division of Experimental Animal Research, Chiba Cancer Center Research Institute, 666-2 Nitonacho, Chuo Ward, Chiba, Chiba, 260-8717 Japan; 3 Iwate Medical Megabank Organization, Iwate Medical University, 2-1-1 Nishitokuta, Yahaba-cho, Shiwa-gun, Iwate 028-3694 Japan; 4 Department of Gene Mechanisms, Graduate School of Biostudies, Kyoto University, Yoshida-Konoe-cho, Sakyo-ku, Kyoto 606-8501 Japan

## Abstract

Recent years have witnessed substantial progress in understanding tumor heterogeneity and the process of tumor progression; however, the entire process of the transition of tumors from a benign to metastatic state remains poorly understood. In the present study, we performed a prospective cancer genome-sequencing analysis by employing an experimental carcinogenesis mouse model of squamous cell carcinoma to systematically understand the evolutionary process of tumors. We surgically collected a part of a lesion of each tumor and followed the progression of these tumors *in vivo* over time. Comparative time-series analysis of the genomes of tumors with different fates, i.e., those that eventually metastasized and regressed, suggested that these tumors acquired and inherited different mutations. These findings suggest that despite the occurrence of an intra-tumor selection event for malignant alteration during the transformation from early- to late-stage papilloma, the fate determination of tumors might be determined at an even earlier stage.

## Introduction

Cancer is a result of genomic disorders represented by DNA mutations that typically lead to loss of DNA repair function and gain of abnormal proliferation function. Numerous reports on the process of malignant alterations suggest that benign tumors progress in a stepwise fashion while acquiring driver and passenger mutations, which eventually invade surrounding tissues to finally migrate to distant tissues^1–6^. The consortium projects represented by The Cancer Genome Atlas (TCGA) have catalogued the main cancer driver mutations and identified diverse driver genes from an identical cancer type as well as from more than 60 primary sites^5^. Since these driver genes promote cancer progression by conferring cells with abnormal biological functions such as limitless proliferation and neo-angiogenesis^3,4^, they are regarded as candidate therapeutic targets. However, the high variation of driver genes within an identical cancer type reflects not only the inter-tumor heterogeneity but also the difficulty of cancer therapy^3,7^. Moreover, recent studies focusing on the intra-tumor environment have suggested a polyclonal structure of tumors due to genomic instability^6–8^. Although tumors initially form from a single cell type, as each tumor cell randomly acquires somatic mutations and then proliferates, the polyclonal cell population is formed based on the different genetic backgrounds among tumor cells^6–8^. This genetic diversity of tumor cells generates the physiological diversity and differences in therapeutic sensitivity among tumor cells. Accordingly, the polyclonal structure of tumors is considered to be the most critical cause of treatment resistance and the recurrence of cancer^6–8^.

More recently, the polyclonal structure of tumors has been addressed under the field termed “intra-tumor heterogeneity”, which has emerged as an essential aspect required for disclosing the entire landscape of tumor progression and delineating the specific causes of resistance to cancer treatment. To best understand the full spectrum of intra-tumor heterogeneity, evolutionary analysis has been performed using multi-region samples, which are obtained from multiple sites of a single malignant tumor, and/or the primary and metastatic tumors from the same individual; accordingly, several models have been proposed to explain the process of tumor progression and the origin of tumors^9–12^. An adaptive (Darwinian) tumor progression model was suggested by which only certain sub-groups (i.e., sub-clones) that could gain advantageous traits to survive would remain in the tumor environment. In the process of stepwise cancer progression, tumors have to overcome several barriers such as the lack of nutrients, immune response from the surrounding tissues, and lack of growth space, among others^4^. These barriers impose a type of selection pressure for tumor cells so that only those that are best adapted to the given tumor environment will survive to proliferate, and will thus acquire various mutations in the process to lead to intra-tumor heterogeneity that contributes to the diversity in treatment sensitivity and sustainable progression of tumors^6–8,11^. In contrast, the neutral evolution theory of tumor progression proposes that tumor cells are derived from an initial malignant cell such as a cancer stem cell that neutrally expands with random mutations, thereby resulting in intra-tumor heterogeneity^11,13^.

Moreover, several hypotheses have been proposed to explain the origin of tumors, including a founder cancer stem cell that already possesses multiple driver mutations and then rapidly grows and forms a tumor via the acquisition of new driver (trigger) mutations, or development of an initial driver mutation that causes an undetectable tumor, which gradually grows in size owing to the acquisition of new driver mutations^12^. Despite these advances in the general understanding of inter-/intra-tumor heterogeneity and the process of tumor progression, the entire evolutionary process of tumors, from a benign to metastatic state, is still poorly understood. Gaining a detailed understanding the process of tumor evolution over time is expected to improve the confidence of early diagnostics and prognostic predictions. However, to date, the majority of cancer studies use specimens that have already transitioned to malignancy, and research based on tracking the transitions from an early benign tumor to a metastatic tumor is relatively limited.

Given this background, we have begun to address this issue with a prospective cancer study with the goal of systematically understanding the evolutionary process of tumors. Toward this end, we have employed an experimental carcinogenesis mouse model, which promotes the formation of squamous cell carcinoma (SCC) on the back skin of the mice. We performed a classical two-stage carcinogenesis protocol to chemically induce the SCCs for mice using 7,12-dimethylbenz(a)anthracene (DMBA) and 12-O-tetradecanoylphorbol-13-acetone (TPA). In essence, we surgically collected a part of a lesion of each papilloma that formed, and followed the progression of each of these tumors *in vivo* over time. These tumors of the same descent were repeatedly sampled at different growth stages to implement a time-series analysis from a benign to metastatic state. During this sampling process, we observed various fates of the tumors: some became malignant, others remained benign, whereas others regressed. Therefore, we decided to focus on these intra-tumor changes over time as well as on the inter-tumor differences according to comparison of tumors with different fates. To achieve a mechanistic understanding of these differences, we performed high-throughput deep-targeted genome sequencing for two tumor series that were collected from the same mouse, one that eventually became malignant and another that regressed, and conducted comparative time-series analysis to understand the evolutionary process of tumors and the factors that determine tumor fates.

SCC is one of the most common cancers in Caucasian populations, and the prognosis of metastatic SCC is extremely poor^14^. The DMBA-induced SCC model is one of the most commonly used *in vivo* models and is widely used for studying the mechanisms of metastasis^15^, which can be applied to both human and mouse cancers^14,16^. High-throughput genome sequencing analysis has previously been conducted for DMBA-induced mouse skin SCCs^17,18^. Nassar *et al*.^17^ investigated the mutational landscape of DMBA-induced SCCs to reveal the substitution patterns of the DMBA-induced somatic mutations and the genes recurrently mutated in this experimental model. McCreery *et al*.^18^ performed phylogenetic analysis for the benign, malignant, and metastatic tumors obtained from the same mouse to reveal the origins of metastases and to indirectly predict the process of tumor evolution. Although these studies reported significant results, almost all of the DMBA-induced SCCs were harvested from metastatic tumors and premalignant tumors at the same time when the mice were sacrificed. Although this sampling method still enabled analyzing tumors at different malignant stages and associating the primary SCC samples with metastatic samples, associations of the premalignant samples could not be determined because their origins are different from those of the SCC samples. Therefore, these previous studies could not eliminate the influence of inter-tumor heterogeneity and could not follow the tumorigenesis process of the same tumor over time. In addition, these previous studies performed whole-exome sequencing and obtained approximately 50× mean coverage sequencing data, whereas Shin *et al*.^19^ indicated that approximately 100× sequencing coverage is required to detect mutations possessed by ~10% of cells in a tumor from clinical samples. Moreover, the known cancer-related gene mutations have been detected with low penetrance in tumor samples due to the influence of intra-tumor heterogeneity^2,9^ and low tumor content^19^. Thus, deep sequencing coverage is necessary for comprehensive mutation analyses; however, the previous data are insufficient to detect low-penetrance variants and to estimate the penetrance in a tumor precisely. With regard to these limitations, our approach, involving partial surgery and targeted deep sequencing, can contribute to gaining a more comprehensive understanding of the tumorigenesis process in different aspects from those obtained in previous research.

## Results and Discussion

### Mutational landscape of two tumor series with different fates

**Figure 1:**
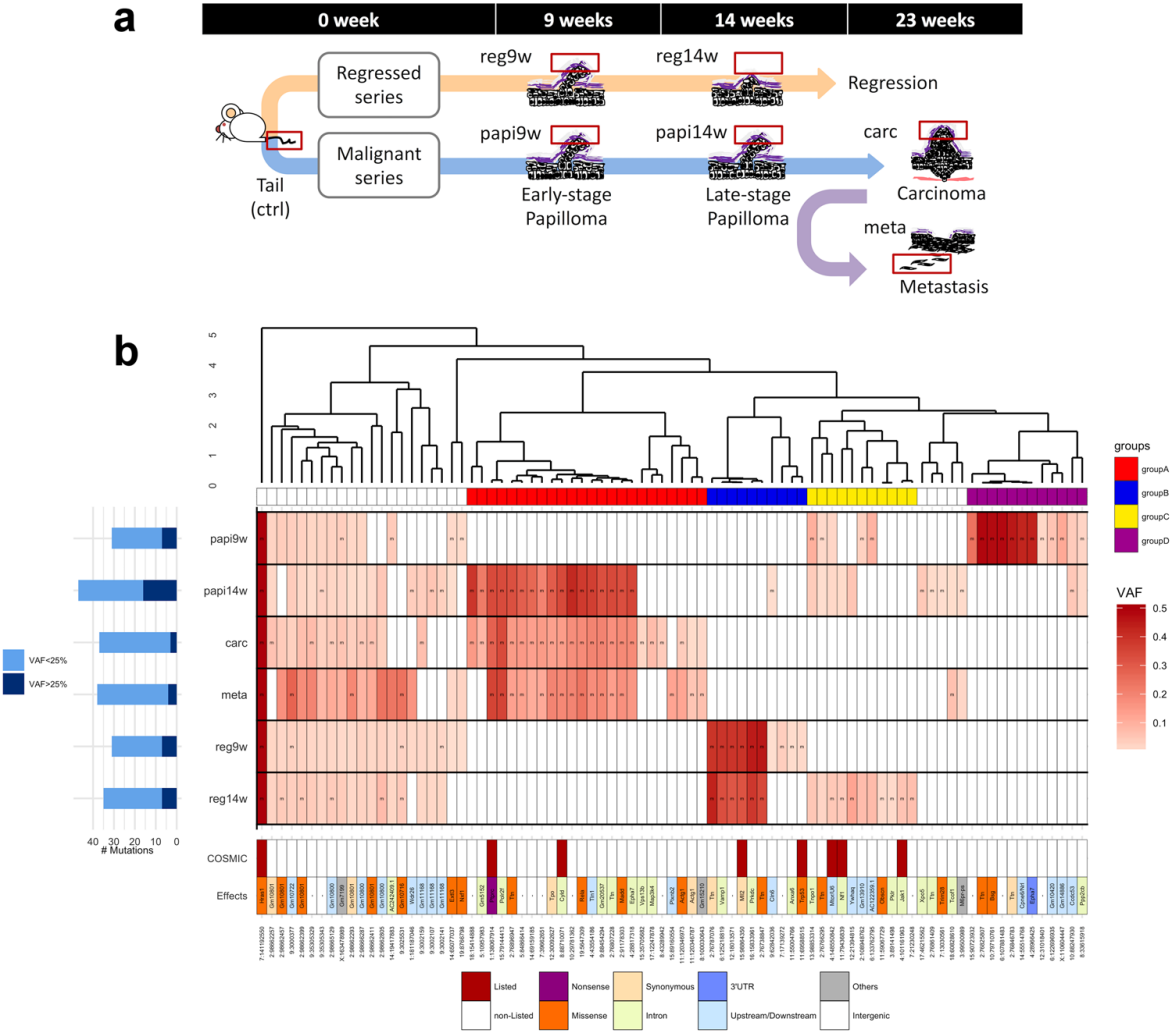
Individual mouse of interest and landscape of the mutational status. (**a**) The mouse developed two tumor series: one eventually regressed and the other eventually metastasized, designated “regressed series” and “malignant series”, respectively. For simplicity, each sample was named as shown near the respective images. (**b**) The overall mutational status of 83 genomic positions for each tumor sample. The bar plot shows the number of positions for which mutant alleles were detected. In the tables on the right, each column corresponds to a certain position. The upper table shows the heatmap of the VAFs with the hierarchical tree color-coded by the mutation groups; “m” denotes that the position was detected by the mutation callers as an SNV. The lower table shows the annotation of the mutations: the first line indicates whether the mutated position is included in a gene listed in the COSMIC: Cancer Gene Census^26^, and the second line indicates the gene name, including the mutated position and the effect of the mutation.

We established the experimental carcinogenesis model using 35 mice for sample collection. One of the main features of the chemical induction mouse skin cancer model is that a large number of benign tumors are formed in the early term, only a few tumors ultimately become malignant^20^. We previously reported that DMBA-TPA carcinogenesis induction forms approximately 30 papillomas on the back skin of each mouse^21^. In the present study, we selected 5–10 DMBA-induced papillomas for each mouse to follow the progression over time. The size and position of each papilloma was recorded during the carcinogenesis experiment, and a part of the papilloma lesion was surgically collected to monitor the progress of the same tumor over time. This partial tumor sampling was performed from the early stage of tumor formation at the ninth week after starting the carcinogenesis experiment. By the time all of the mice were sacrificed, a total of 113 tumors were collected at multiple time points: 8 (7.1%) tumors became malignant, 68 (60.2%) tumors were still benign, and the other 37 (32.7%) tumors had eventually regressed. To understand the differences between the tumors that eventually metastasized and those that eventually regressed, we performed a comparative time-series analysis for two prospectively sampled tumor series obtained from the same mouse: one was sampled at four time points and had eventually metastasized (hereafter “malignant series”), and the other was sampled at two time points and eventually regressed (hereafter “regressed series”). Conceptual figure of tumor samples is shown in Fig. 1a (Supplementary Fig. S1 for photographic images). For simplicity, each sample was named as described in the respective images shown in Fig. 1a. We collected axillary lymph node metastases as metastatic tumor samples, and a matched tail sample was used as a control tissue.

For sensitive detection of mutant alleles from the heterogeneous tumor samples and for accurate estimation of the penetrance of each mutation in the tumors, a capture sequencing approach was adopted, which can achieve deep-sequencing coverage for targeted genes (see Supplementary Table S1 for the list of targeted genes and see Supplementary Table S2 for sequencing statistics). The single-nucleotide variants (SNVs) were detected using three variant callers and then filtered according to several criteria to minimize false positives. We defined candidate mutation positions as the positions at which SNVs were detected in at least one tumor sample, and focused on the 83 candidate mutation positions with >50× coverage in all seven samples (see Supplementary Table S3 for the sequencing depth of these positions). Next, the variant allele frequencies (VAFs) were calculated for each of the 83 candidate mutation positions in each tumor sample. The VAF is a percentage of variant alleles in the short reads that are mapped to a certain genomic position; therefore, it can be considered as an index of the penetrance of a mutation in a tumor, and can serve as a clue to infer the intra-tumor heterogeneity and sub-clonal structure in tumors^2,13^. The VAFs of the positions with less than three reads supporting the mutation were filtered out (set to 0), and were used to estimate the tumor purity for each tumor sample.

The heterozygous Q61L *Hras* mutation (chr7:141192550) is known as an initiator of the DMBA-TPA experimental carcinogenesis protocol we employed^17,18,22^; therefore, the tumor purity was estimated based on the VAF of the *Hras* mutation (Supplementary Table S4). Correspondingly, the raw VAFs were normalized to the estimated tumor purities for proper estimation of the penetrance of the mutations in the tumors while excluding the influence of normal cell-derived reads. Finally, the candidate mutation positions with a normalized VAF ≠ 0 were presumed to be the mutated positions for each tumor sample.

Figure 1b shows the overall mutational status for the 83 candidate mutation positions in each tumor: the annotation of mutations, heatmap of the normalized VAFs with the hierarchical tree, and number of mutated positions are shown (see Supplementary Table S5 for more details). First, the number of mutated positions tended to increase during the benign stages in both tumor series, but decreased during the malignant stages. This indicates that the cells in the tumor acquired new mutations during the benign state, whereas the cells with several private mutations could not survive after malignancy, suggesting a selective process of malignant alteration.

Next, we evaluated the commonality of the mutations detected between the tumor types to better understand the nature of the inter-tumor heterogeneity. From the heatmap with a hierarchical tree shown in Fig. 1b, 9/83 (10.84%) mutations were observed in all tumor samples, and the other 74/83 (89.16%) mutations were privately observed or sparsely shared among tumors. There were mutation groups inherited in each tumor series; in particular, two mutation groups were mutually exclusively detected in each tumor series (groups A and B): the mutations belonging to group A were observed after 14 weeks in the malignant tumor series, whereas the mutations belonging to group B were observed only in the regressed tumor series. These mutation groups might reflect the sub-clonal structure in the tumors. Specifically, an intronic mutation of *Cyld* and a nonsense mutation of *Ptprc* were included in mutation group A. The *Cyld* gene is known as a deubiquitinase whose deubiquitination activity negatively regulates the NF-κB pathway and suppresses epidermal tumor progression^23,24^. In addition, *Ptprc* encodes CD45, which regulates the phosphorylation of SRC and JAK family members^25^, and nonsense mutations in this gene have been recurrently reported in human epithelial and blood cancers (http://cancer.sanger.ac.uk/cosmic). Moreover, both of these genes are listed in the COSMIC: Cancer Gene Census (CGC)^26^, a catalogue of cancer-related genes. These two genes showed a high penetrance rate in the tumor samples of the malignant tumor series at the later stages (Fig. 1b), suggesting that inactivation of these genes caused by mutations could have driven the tumor progression.

Moreover, a synonymous mutation in *Mll*2 and a missense mutation in *Trp53* were included in mutation group B. Mutations of these genes have been reported to promote cancer progression^26^, especially mutation of the *Trp53* tumor suppressor gene, which has been widely reported as a driver mutation in several cancer types^3,27^. *Trp53* is a well-known tumor suppresser gene that plays an important role for DNA repair function, cell cycle arrest, and apoptosis^28^. *Mll*2 is a histone methyltransferase and its mutants provoke the genomic instability in tumors^29^. There are reports that the *Trp53* mutations induce not only abnormal proliferation in tumors but also anti-tumor effects^28,30^, and these reports had also discussed the influence of the *Trp53*-independent apoptosis. Therefore, we considered the possibility that these mutations, in *Trp53* and *Mll*2 genes, which are related to genomic instability, could have induced the *Trp53*-independent apoptosis, which in turn resulting in the eventual regression of the tumor.

Another mutation group, group C, showed an opposite pattern to that of the other two tumor series. The tumor of the malignant series acquired group C mutations when it was benign, but did not inherit these mutations after malignant transformation. By contrast, the tumor of the regressed series acquired the mutations at a later stage of the benign term. The mutations of genes listed in the CGC as cancer-related genes, although in non-exonic regions, were enriched in group C. For instance, downstream mutation of *Mtor* and intronic mutations of *Nf1* and *Jak1* were included in the group C, and these genes have a crosstalk and/or direct regulatory relationship with the RAS signaling pathway^31–33^. In comparison, group D represented a mutation group that was observed only in the early stages of the malignant tumor series, and none of the included gene mutations are listed in the CGC. This suggests that the peripheral genomic region of cancer-related genes was more actively mutated in the regressed tumor series. Furthermore, focusing on the malignant tumor series, the mutations showing relatively high VAFs in the 9^th^ week papilloma group (group D) were not detected at the later stages, and the mutations conserved after the 14^th^ week of papilloma development (group A) were not detected in the 9^th^ week; hence, it appears that the 9^th^ to 14^th^ week represents a period of substantial changes in the intra-tumor sub-clonal structure for malignant alteration. Thus, the mutational status among tumors suggests that the sub-clonal structure in the tumors continuously changes over time and also differs between tumors with different fates.

Furthermore, to evaluate the adequacy of these results, we reviewed previous studies using the same mouse carcinogenesis protocol. Nassar *et al*.^17^ and McCreery *et al*.^18^ performed large-scale analyses of SCC induced in the skin of mice with DMBA-TPA, and identified recurrent mutations among the SCCs, including exclusive driver mutations of the RAS family, and revealed the mutational signature of DMBA induction enriched with A > T base substitutions. Consistently, in our results, all of the tumors had the Q61L *Hras* mutation, which was reported as the initial driver mutation for this model^17,18,22^, and the substitution pattern of the 83 candidate mutation positions showed enrichment for the A > T base substitution (Supplementary Fig. S2); therefore, our results are in line with previous studies of this model.

Of note, the previous studies identified a recurrent mutation in the *Trp53* gene in the malignant SCCs, whereas this mutation was only detected in the early stage of the regressed tumor series in the present study. This suggests that a single *Trp53* mutation may not promote tumor progression. By contrast, the stop-gain mutation of *Ptprc*, which belongs to the protein tyrosine phosphatase (PTP) family, was inherited in the malignant tumor series. Nassar *et al*.^17^ also identified another gene belonging to the PTP family, *Ptprm*, that showed recurrent mutations; hence, the PTP family appears to play a key role in the DMBA-TPA experimental carcinogenesis system.

The results highlighted thus far exhibited an increase in the number of mutations while the tumor is in the benign stage with a subsequent decrease in mutations after malignant alteration. The tumor series with different fates, i.e., becoming malignant and eventually regressing, acquired and inherited different mutations; however, not all mutations were inherited, and the VAFs also changed over time. These results suggest that selection pressure was applied to induce the process of malignant alteration, and the sub-clonal structure not only differs between tumor fates but also changes over time.

### Degree of shared mutations between tumors within and among tumor series

**Figure 2:**
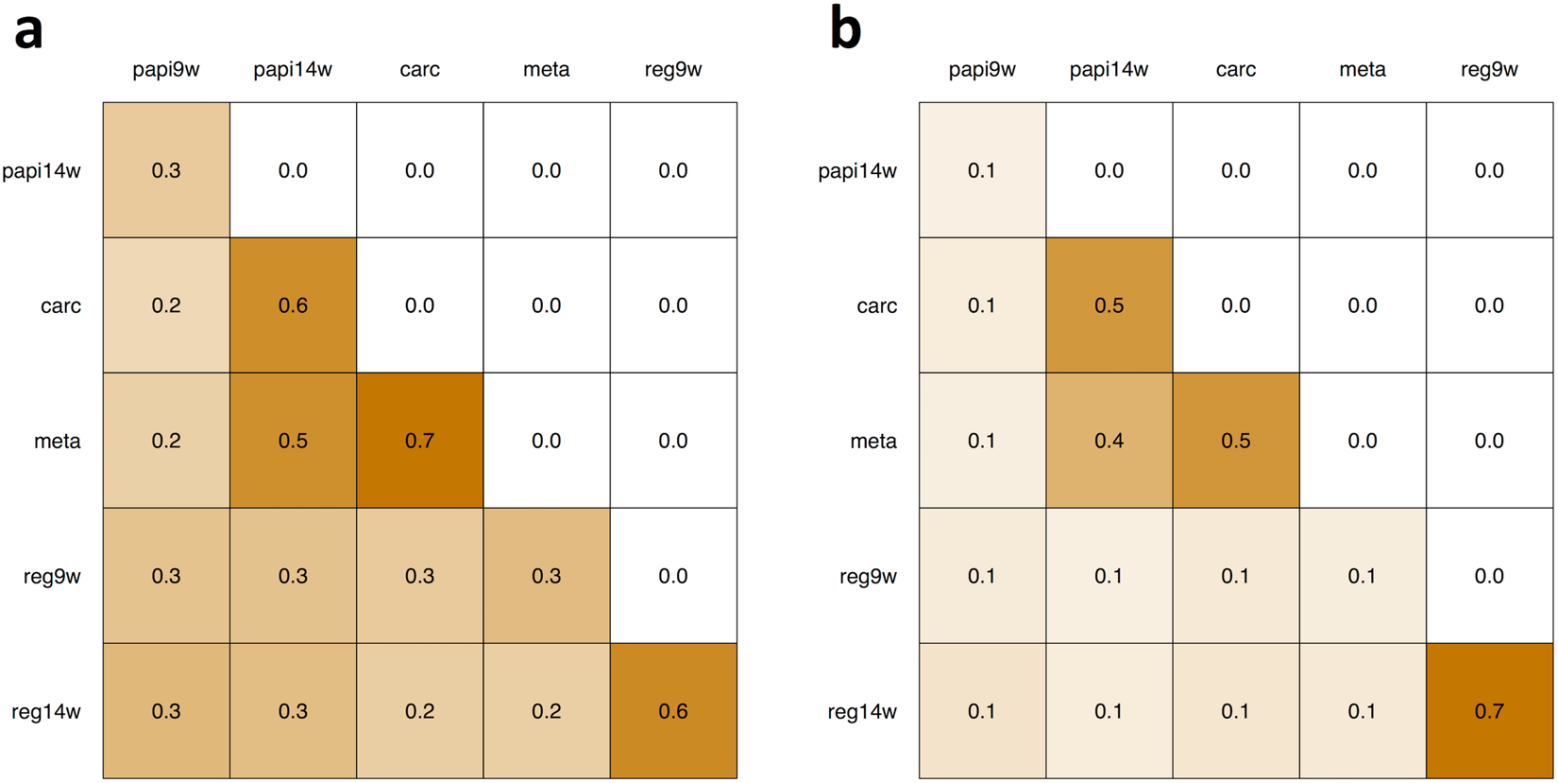
Heatmap of similarity between tumors. (**a**) Heatmap of the Jaccard similarity coefficient between tumor samples. (**b**) Heatmap of the generalized Jaccard similarity coefficient between tumor samples.

Subsequently, we evaluated the similarity (or dissimilarity) between tumors by applying the Jaccard similarity coefficient and the generalized Jaccard similarity coefficient (Fig. 2). For this analysis, we assumed that the positions with a VAF ≠ 0 of the 83 candidate mutation positions were mutated, and defined a set of mutations for each tumor sample. To determine the commonality of the mutated positions between tumors, we calculated the Jaccard similarity coefficient between the sets of mutations. The heatmap of the Jaccard similarity coefficient (Fig. 2a) indicated that the similarity between tumors within the same tumor series was higher than that between tumor series; however, the similarity between the 9^th^ week and later-stage papilloma samples of the malignant tumor series was only moderate. This result supports that different mutations are inherited in each tumor series, and confirms that the intra-tumor structure existing in the malignant tumor series dramatically changed between the 9^th^ to 14^th^ weeks, as proposed from the results of the mutational status analysis (Fig. 1b).

Moreover, we applied the generalized Jaccard similarity coefficient to quantitatively evaluate the inheritance of the mutations. We used the normalized VAF value to represent the penetrance of a mutation in a tumor, and defined a numeric vector constructed by the normalized VAFs of the 83 candidate mutation positions for each tumor sample. The generalized Jaccard similarity coefficient between these numeric vectors then corresponds to the commonality of the abundances of the mutations between tumors. The heatmap of the generalized Jaccard similarity coefficient between tumors (Fig. 2b) not only accentuates the same features represented in Fig. 2a but also indicates that the penetrance of the mutations was more highly conserved between tumors of the regressed tumor series than that between tumors of the malignant tumor series. Consistently, the scatter plot of the normalized VAFs between tumors (Supplementary Fig. S3) showed that the plots between tumors with relatively low generalized Jaccard similarity coefficients were clearly biased toward both axes, whereas the plots between tumors with higher similarity coefficients, especially those between tumors within the regressed tumor series, basically lay on the diagonal line. These results suggest that tumors of the regressed series were relatively stable over time, whereas the intra-tumor structure of the malignant tumor series was crucially changed between the 9^th^ to 14^th^ weeks, and then continuously changed gradually after the 14^th^ week.

### Intra-tumor heterogeneity and evolutionary process within tumors

Next, we focused on the intra-tumor heterogeneity. To evaluate the degree of intra-tumor heterogeneity, we applied an entropy parameter for the normalized VAFs of the 83 candidate mutation positions. Entropy is often used as an index of the complexity or information content of certain events. Dr. Shannon proposed an expected value of the information amount as “information entropy”^34^, and this concept has been widely applied in the fields of informatics, statistics, and biology^35^. In our study, we regarded the intra-tumor heterogeneity to reflect the complexity of the tumor, which was evaluated by the sum of the complexity calculated for each mutation.

**Figure 3:**
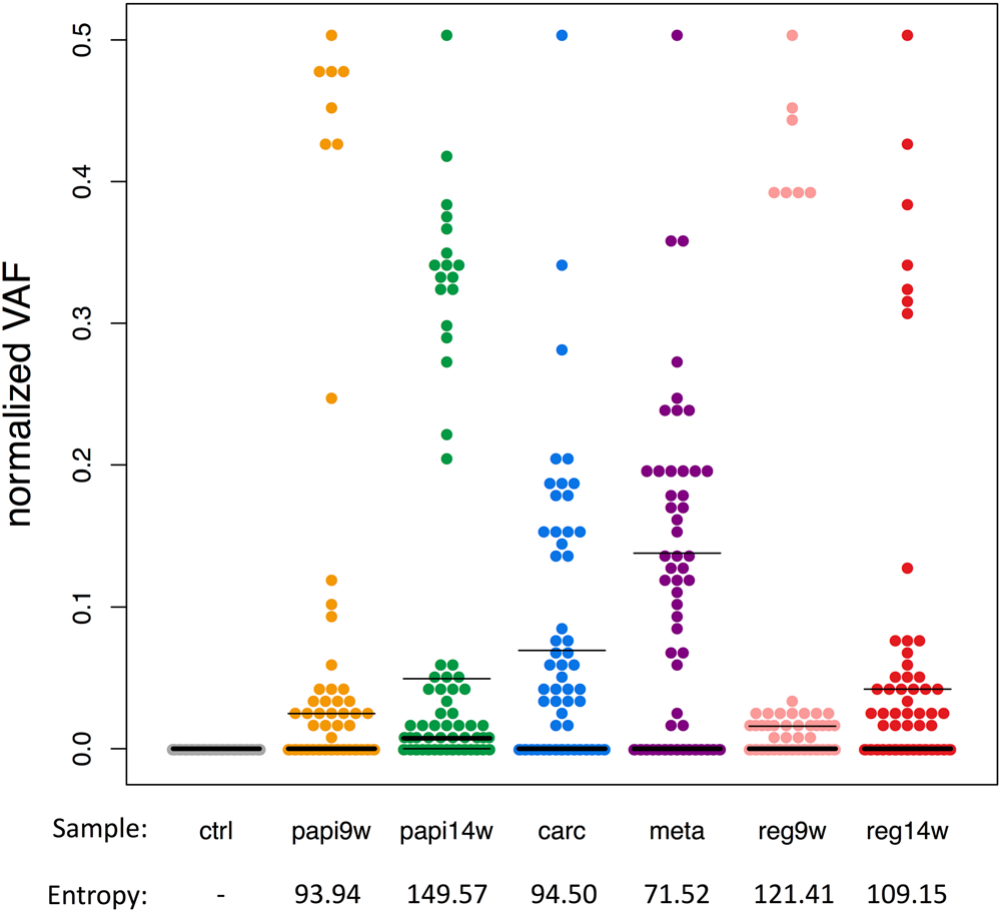
Spectrum of normalized VAFs and entropy of each tumor sample. The chart shows the spectrum of the normalized VAFs, where each column represents a certain sample, and the y-axis corresponds to the normalized VAF. Each dot corresponds to a certain candidate mutation position for a total of 83 dots plotted for each column. The values shown at the bottom of each column denote the entropy parameter values.

As shown in Fig. 3, the spectrum of the normalized VAFs demonstrated that all normalized VAFs were below the 50% line (the plot on the 50% line in each of the tumor samples corresponds to the *Hras* mutation). The genomic positions with a normalized VAF of 0% indicates that the position is homozygous to that of the reference base in all tumor cells, while the positions with a normalized VAF of 50% indicates that the position is heterozygous in all tumor cells; in other words, these positions are homogeneous among tumor cells. Based on this result, we assumed that there was no copy number variation (CNV) at the 83 candidate mutation positions and that these mutations occurred on one side of the diploids. Moreover, the clonal structure of a tumor can be represented by a combination of mutations; therefore, we assumed that the mutations are independent but do not contradict each other. With these assumptions, we defined an entropy parameter as the degree of complexity of a tumor (see Materials and Methods for more details), which was calculated from the normalized VAFs of the heterogeneous positions for each tumor sample (Fig. 3, bottom). As a result, the entropy was found to be maximal in the 14^th^-week papilloma sample, and then decreased after the tumor became malignant in the malignant tumor series. Moreover, the entropy value of the regressed tumor series decreased over time, and was lower than that of the 14^th^-week papilloma sample of the malignant tumor series. These results suggest that the tumors of the malignant series gradually acquired intra-tumor heterogeneity before developing malignancy, whereas the tumors of the regressed series could not sustain the heterogeneous characteristic.

**Figure 4:**
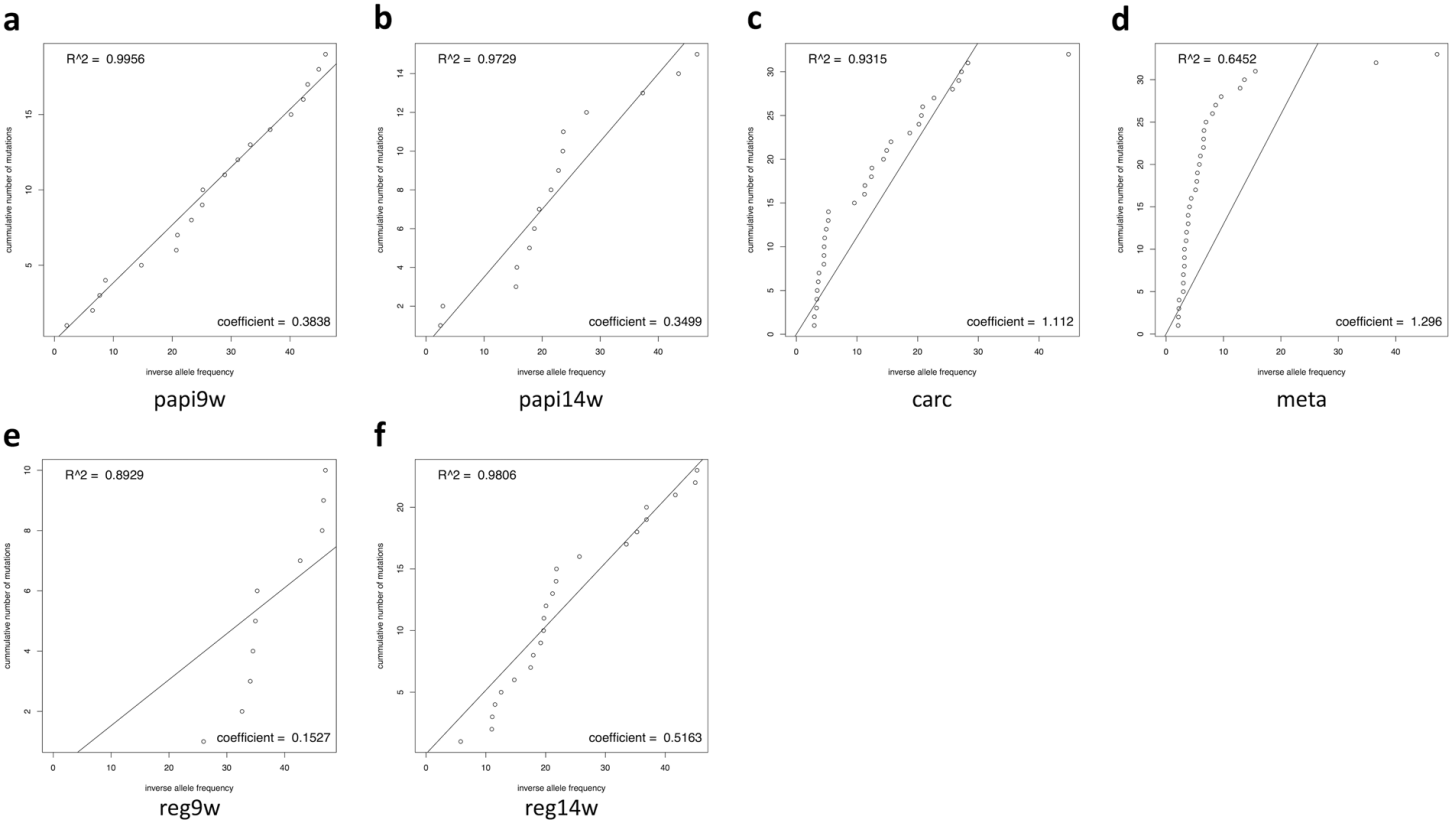
Mutation rate analysis for the malignant tumor series (**a**–**d**) and the regressed tumor series **(e**,**f**). The apparent mutation rate is shown at the right bottom of each plot, and the coefficient of determination (*R*^*2*^) is shown at the left top of each plot.

Furthermore, we estimated the mutation rate to obtain insight into the process of sub-clonal evolution in the tumors. Under the assumption that mutations are neutrally accumulated and penetrate a tumor over time, the number of mutations should proportionally increase with time and the mutation rate of a tumor cell should be constant. Williams *et al*.^13^ proposed a regression analysis method to estimate the mutation rate from VAFs. According to their results, we assumed that the position with a normalized VAF of 25% or less in the 83 candidate mutation positions represented a sub-clonal mutation. For each tumor sample, the sub-clonal mutations were sorted in ascending order according to the inverse of the normalized VAFs, and the normalized VAFs were plotted against these sorted ranks, which were used to perform the regression analysis respectively for the ranks (Fig. 4). The slope of the regression line denotes the estimated apparent mutation rate (shown at the right bottom of each plot), and the coefficient of determination (*R*^*2*^) denotes the neutrality of the mutations (shown at the left top of each plot). When *R*^*2*^ ≥ 0.98, the tumor was considered to have acquired mutations neutrally; otherwise, the tumor was considered to have acquired mutations in a non-neutral manner (i.e., selectively). The mutation rates of the benign tumors were lower than those of the tumors that transitioned to a malignant status; this effect was particularly notable in the 9^th^ week early-stage papilloma sample of the regressed tumor series. For the malignant tumor series (Fig. 4a–d), the mutations were neutrally acquired until the 9^th^ week papilloma, but were then acquired in a biased manner after the 9^th^-week papilloma; notably, an early selection mode^13^ was suggested for carcinoma and metastasis progression, indicating that the tumor had already gained dominant (selected) mutations (sub-clones) before transitioning to carcinoma. In brief, the tumor was exposed to the selection pressure after 9 weeks before becoming malignant. According to the mutational landscape (Fig. 1b) and the similarity between tumors (Fig. 2), the intra-tumor structure of the malignant tumor series was dramatically changed between the 9^th^ and 14^th^ weeks, and then was comparatively conserved thereafter. Thus, the selection pressure that had been in effect from the 9^th^ to 14^th^ weeks might have caused the dramatic changes in the tumor. By contrast, for the regressed tumor-series (Fig. 4e,f), the tumor had already been acquiring the mutations selectively before the 9^th^ week; however, the process of mutation acquisition after the 9^th^ week was comparatively neutral. These results suggest that different tumor series undergo distinct evolutionary processes, indicating that the tumor fate is determined from a quite early stage.

## Conclusion

In this study, we compared tumor series with different fates through analysis of a DMBA-TPA carcinogenesis protocol. Although the DMBA-TPA protocol is limited to the analysis of the tumors initiated by RAS family mutation, it is widely accepted that the mutation landscape, mutation burden, and gene expression profile of the SCCs formed on the mouse back skin through the DMBA-TPA protocol are largely similar to human SCCs such as head and neck, esophageal, lung, and cervical SCCs^14,17,18,36,37^. In line with these previous studies, our results indicated that although the carcinogenesis protocol using DMBA-TPA provides information on the initial driver mutations and promotes the formation of a number of papillomas on the mouse skin, further drivers are clearly required for the malignant alteration. In most cases, papillomas could not become carcinomas, meaning that further driver events did not occur, while also confirming the general difficulty of malignant transformation. We further presumed that there are differences in the genomic mutations and in the sub-clonal structure among tumors with different fates. To understand the difference between the eventually metastasized and regressed tumors, we performed targeted deep sequencing for these two tumor series of different destinies sampled from the same mouse. This comparative analysis provided an opportunity to understand the factors and timing of the tumor fate determination.

Previous cancer studies reported that each tumor has a different mutational status and multiple driver mutations are accumulated during tumor progression^3,5,12,26^. To understand the evolutionary process of tumors, the temporal changes of the mutational trend, e.g., the order of the occurrence of driver events, have also been discussed^1–3,10,11,38,39^. These studies identified that known driver mutations were significantly biased to be clonal; hence, it has been considered that these driver mutations are acquired before malignant transformation, and the highly malignant sub-clones with these driver mutations originate to form malignant tumors^6,12^. Consistent with these previous hypotheses, our results suggest that tumors with different fates acquired different mutations, accompanied by changes in the intra-tumor structure over time, and also suggest that selection pressure could have influenced the benign tumors to select highly malignant sub-clones in the tumors.

Moreover, our results provide new insight into the timing of the selective event and fate determination of tumors. The selection event for malignant alteration might have occurred between the 9^th^ and 14^th^ weeks, corresponding to the transformation from early-stage papilloma to late-stage papilloma, and the evolutionary process of these two tumor series differed at the time of the 9^th^ week early-stage papilloma. Furthermore, only the tumors that could obtain and sustain sufficient intra-tumor heterogeneity could become malignant. The majority of cancer studies conducted to date have focused on malignant tumors^5^. Although several studies analyzed tumors at both the benign and malignant stages, these tumor types are typically collected from different patients^10^, or the adjacent lesions of primary tumors were collected as precursor lesions^39^; therefore, these studies could not follow the temporal changes of the same tumors respectively. Thus, our prospective approach is complementary to existing cancer studies, and offers novel insights.

Adoption of this prospective study model can provide new opportunities for gaining a detailed understanding of the evolutionary process of tumors by conducting statistical analysis with increased numbers of mice and tumor series, and also by analyzing the whole genome rather than targeted regions. Moreover, our study can also be relevant to improving surgical treatment resistance and prognostic prediction^40^. In particular, our results and further statistical analysis with more mice can help to identify factors that determine the fate of tumors and the prognostic biomarkers for guiding a more precise treatment strategy. However, the gene signatures and the timeline of tumor progression might vary in different tumor samples; therefore, further experiments are required to verify these aspects. As part of our future works, we are planning to perform whole-exome sequencing for more tumor series, and other omics analyses such as transcriptomics and epigenomics.

## Methods

### Mice

This study was conducted in strict accordance with the recommendations in the Guide for the Care and Use of Laboratory Animals of the Ministry of Education, Culture, Sports, Science, and Technology of Japan. The protocol was approved by the Committee on the Ethics of Animal Experiments of Chiba Cancer Center (Permit Number: 17–14). All efforts were made to minimize suffering. FVB/N mice were purchased from CLEA Japan (Tokyo, Japan).

### Skin carcinogenesis and tumor sampling

DMBA was used as the carcinogen and TPA was used as a promoter to induce SCC on the back of the mouse skin. We treated 35 FVB/N mice according to a two-stage carcinogenesis protocol. At 8 weeks of age, the female mice were carefully shaved with an electric clipper, and two days after shaving, a single dose of DMBA (25 μg/mouse in 200 μL of acetone) was applied to the shaved dorsal back skin. One week after initiation, tumor growth was promoted with TPA (10 μg/mouse in 200 μL of acetone) twice weekly for 20 weeks. For each mouse, 5–10 papillomas were selected to follow tumor progression over time. The number, size (diameter in mm), and position of each papilloma were recorded as of 9, 14, and 23 weeks, and carcinoma development was monitored for up to 33 weeks post-TPA treatment. To obtain the prospective tumor samples from the same tumor, a part (approximately half) of the papilloma lesions was surgically collected so that the remaining part could grow toward the later stages. These collected samples were further cut in half; therefore, one quarter of each tumor was used for this research, and the remaining quarter was preserved for subsequent research. We determined benign tumors (papillomas) and malignant tumors (carcinomas) by visual inspection. Papillomas appear as outgrowths on the mouse back skin. Some of them become flattened on the skin with malignant transformation involving penetration of the deep dermis. In general, these tumors can be easily distinguishable from one another^14,21^, and we did not use any of the tumors that could not be clearly classified as papilloma or carcinoma, such as an intermediate type. The mice were sacrificed by cervical dislocation when carcinoma formed and axillary lymph node metastases were visually confirmed, or when the tumor volume reached 10% of the mouse body weight. We collected axillary lymph node metastases as metastatic tumor samples. As a control tissue, we collected a tail sample from each mouse before starting the DMBA-TPA experiment. Finally, the time-series tumor samples that eventually became malignant and those that regressed were obtained from the same mouse (Fig. 1a and Supplementary Fig. S1).

### Sample preparation and targeted deep sequencing

For sensitive detection of the low-frequent mutant alleles and for precise estimation of the VAFs, we adopted a targeted capture-based deep-sequencing approach. We compiled a list of the target genes by reference to the COSMIC database (http://cancer.sanger.ac.uk)^41^ to select the most frequently mutated genes in the SCC samples, and used previous reports of housekeeping genes^42,43^ to select the genes deemed to be most crucial for cell survival. Finally, we selected 500 genes and designed the target capture bait library using the Agilent SureDesign program (https://earray.chem.agilent.com/suredesign; see Supplementary Table S1 for details).

For sample preparation, the genomic DNA was extracted from the tumors and normal tissues (tail of each mouse) using DNAiso Reagent (9770 A; Takara, Otsu, Japan). The extracts were treated according to SureSelect^XT^ Target Enrichment System for Illumina Paired-End Sequencing Protocol for 200-ng samples (Agilent Technology, Santa Clara, CA, USA). In this step, pre-capture polymerase chain reaction (PCR) was performed for 10 cycles, the incubation for library hybridization was performed for 24 h, and post-capture PCR was performed for 12 cycles. The products were confirmed using the Agilent 2100 bioanalyzer. The paired-end sequencing (2 × 75 bp) was performed using the Illumina MiSeq system with MiSeq Reagent Kit v3 (150 cycle; Illumina, San Diego, CA, USA).

### Sequence data analysis

The sequencing adapters and low-quality ends of raw sequenced reads were trimmed using Trimmomatic (version-0.36)^44^ with the options “ILLUMINACLIP:${adapter.fa}:2:30:10 LEADING:30 TRAILING:30 SLIDINGWINDOW:4:25 MINLEN:1”, and the low-quality reads were filtered using FASTX-Toolkit (http://hannonlab.cshl.edu/fastx_toolkit/index.html) with the options “-q 30 and -p 80”. Next, the remaining paired reads were mapped to the mouse reference genome (GRCm38-release71.fa) using the BWA-MEM algorithm^45^ (Burrows-Wheeler Aligner; BWA; version-0.7.15-r1140). The reads with a mapping quality under 25 were filtered, and the others were treated according to the Genome Analysis Toolkit (GATK) best practices (de-duplicates, base recalibration, and local realignment) using GATK^46^ (version 3.6), picard tools (http://broadinstitute.github.io/picard; version 2.6.0), and samtools^47^ (version 1.3.1). The SNVs were detected by MuTect2^48^ (within GATK 3.6; default settings), Strelka^49^ (version 1.0.14; skip depth filters to follow the recommendation), and LoFreq.^50^ (version 2.1.2; default settings with 0.01 significance level and Bonferroni correction). Among these unions of detected SNVs, the SNVs corresponding to any of the following cases were excluded: (a) registered in the dbSNP^51^ (release version 3), (b) supported by <3 reads, (c) reads supporting mutant alleles mapped in the control sample, and (d) other SNVs detected within 10 bp on either side. We defined candidate mutation positions as the positions detected to be SNVs in at least one tumor sample, which clearly passed above the filters, and we focused on 83 candidate mutation positions whose sequence coverage was >50× in all seven samples. Next, we calculated the percentage of variant alleles among the mapped short reads for each candidate mutation position from the mapped read bases whose Phred quality score was >13. The VAFs of the positions for which the number of reads supporting the mutation was less than 3 were filtered out (set to 0). Moreover, we estimated the tumor purity of the tumor samples according to the VAF of the Q61L *Hras* mutation (chr7:141192550), because this heterozygous mutation is known as an initiator of DMBA-TPA experimental carcinogenesis^17,18,22^. Accordingly, we estimated the proper penetrance rates of the mutations in the tumors while excluding the influence of the normal cell-derived reads by normalizing the raw VAFs with the estimated tumor purities. The normalized VAFs were used for subsequent analyses. The sequencing, read mapping, and tumor purities are summarized in Supplementary Tables S2–S4.

### Hierarchical clustering of candidate mutation positions

We assigned a six-dimensional vector constructed by the normalized VAFs of six tumor samples for each of the 83 candidate mutation positions, and performed agglomerative hierarchical clustering. We adopted a weighted Manhattan distance (Canberra distance) approach as the distance measure between the assigned numeric vectors and used the group mean method for clustering. The Canberra distance between numeric vectors ***x*** and ***y*** can be calculated as follows:

1 $$d({\boldsymbol{x}},\,{\boldsymbol{y}})=\sum _{i}\frac{|{x}_{i}-{y}_{i}|}{|{x}_{i}|+|{y}_{i}|}\,$$

### Similarity measurements between tumors

To evaluate the similarity between tumors, we applied the Jaccard similarity coefficient and generalized Jaccard similarity coefficient. The Jaccard similarity coefficient is a similarity measure representing the degree of commonality between two sets. The Jaccard similarity coefficient between sets *A* and *B* can be calculated as follows:

2 $$J(A,B)=\frac{|A{\cap }^{}B|}{|A{\cup }^{}B|}$$

In our study, we defined a set of mutations for each tumor sample and calculated the Jaccard similarity coefficient between the sets of mutations to represent the degree of similarity between tumors^52^.

In addition, we defined an 83-dimensional numeric vector constructed by the VAFs of the 83 candidate mutation positions, and assigned a numeric vector for each tumor sample. We calculated the generalized Jaccard similarity coefficient between tumor samples using the defined numeric vectors as the similarity between the tumors. The generalized Jaccard similarity coefficient between numeric vectors ***x*** and ***y*** can be calculated as follows:

3 $$gJ({\boldsymbol{x}},{\boldsymbol{y}})=\frac{{\sum }_{i}\,{\rm{\min }}({x}_{i},{y}_{i})}{{\sum }_{i}\,\max ({x}_{i},{y}_{i})}$$

### Evaluation of intra-tumor heterogeneity

To evaluate the intra-tumor heterogeneity, we applied an entropy parameter. We regarded the intra-tumor heterogeneity as the complexity of the tumor, which was evaluated by the sum of the complexity provided by each mutation. From the spectrum of the normalized VAFs (Fig. 3), we assumed that there was no CNV at the 83 candidate mutation positions and that these mutations occurred on one side of the diploids. With this assumption, we defined the probability that a cell randomly selected from a tumor *i* has a mutation *m* as *P*(2 × nVAF_*m,i*_), and defined its self-information amount as −log*P*(2 × nVAF_*m,i*_), where nVAF_*m,i*_ denotes a normalized VAF of a mutation *m* in a tumor T*i*. Moreover, the clonal structure of a tumor can be represented by a combination of mutations; thus, we assumed that the mutations are independent but do not contradict each other. Under these assumptions, we defined an entropy parameter as the degree of the complexity of a tumor according to the following equation:

4 $$H({T}_{i})=-\sum _{m\in {M}_{i}}\,\mathrm{log}\,P(2\times {{\rm{nVAF}}}_{m,i})$$

where *M*_*i*_ denotes a set of candidate mutations in a tumor *T*_*i*_.

### Estimation of the mutation rate and evolutionary process

According to a previously reported method^13^, the mutation rate and evolutionary process of the tumors were estimated from the normalized VAFs. On the assumption that the sub-clonal mutations were neutrally accumulated and penetrated a tumor over time, the number of mutations should proportionally increase with time, and thus the mutation rate of a given tumor should be constant. Therefore, Williams *et al*.^13^ proposed a fitting model to estimate the mutation rate of a tumor and to infer the neutrality of the sub-clonal mutations in a tumor as follows:

5 $$M(f)={\mu }_{e}(\frac{1}{f}-\frac{1}{{f}_{max}})$$

where *f* denotes the relative fraction of a mutation in a tumor, *M*(*f*) denotes the cumulative number of mutations, and *µ*_*e*_ denotes the apparent mutation rate. The *f* value corresponds to the VAF of a mutation, and *f*_*max*_ = 0.5 when assuming a diploid tumor. Under the same assumptions described above, the VAF of a mutation should increase for mutations that occur earlier; hence, the cumulative number of mutations *M*(*f*) can be estimated by sorting the mutations according to the VAFs. Thus, we assumed that the position with a normalized VAF that was less than 25% in the 83 candidate mutation positions was a sub-clonal mutation. To exclude false positives, we eliminated the mutations whose VAFs were below 2% in this analysis. For each tumor sample, the sub-clonal mutations were sorted in ascending order by the inverse of the normalized VAFs, the normalized VAFs were plotted against these ranks, and regression analysis was performed based on equation (5). Finally, the slope of the regression line and the coefficient of determination were calculated, respectively.

## Electronic supplementary material

Supplementary Information

## Data Availability

All sequencing data used in this work are available from the DNA Data Bank of Japan (DDBJ) Sequence Read Archive (DRA) under the accession number DRA007132.
