# Supplementary material for: Time-Series Analysis of Tumorigenesis in a Murine Skin Carcinogenesis Model
Source: Sci Rep. 2018 Aug 29;8:12994. doi: 10.1038/s41598-018-31349-x (PMC6115443; doi:10.1038/s41598-018-31349-x)
Supplement: Supplementary file 1 — Supplementary Information [file 41598_2018_31349_MOESM1_ESM.docx]

Supplementary Information for
“**Time-Series Analysis of Tumorigenesis in a Murine Skin Carcinogenesis Model**”

Yoshimasa Aoto^1^, Kazuhiro Okumura^2^, Tsuyoshi Hachiya^3^, Sumitaka Hase^1^, Yuichi Wakabayashi^2^, Fuyuki Ishikawa^4^, Yasubumi Sakakibara^1*^

^1^Department of Biosciences and Informatics, Keio University, 3-13-1 Hiyoshi, Kohoku-ku, Yokohama 223-8522, Japan, ^2^Department of Carcinogenesis Research, Division of Experimental Animal Research, Chiba Cancer Center Research Institute, 666-2 Nitonacho, Chuo Ward, Chiba, Chiba 260-8717, Japan, ^3^Iwate Medical Megabank Organization, Iwate Medical University, 2-1-1 Nishitokuta, Yahaba-cho, Shiwa-gun, Iwate 028-3694, Japan, ^4^Department of Gene Mechanisms, Graduate School of Biostudies, Kyoto University, Yoshida-Konoe-cho, Sakyo-ku, Kyoto 606-8501, Japan

*Correspondence and requests for materials should be addressed to Y.S. (email: yasu@bio.keio.ac.jp)

**Supplementary Figures**


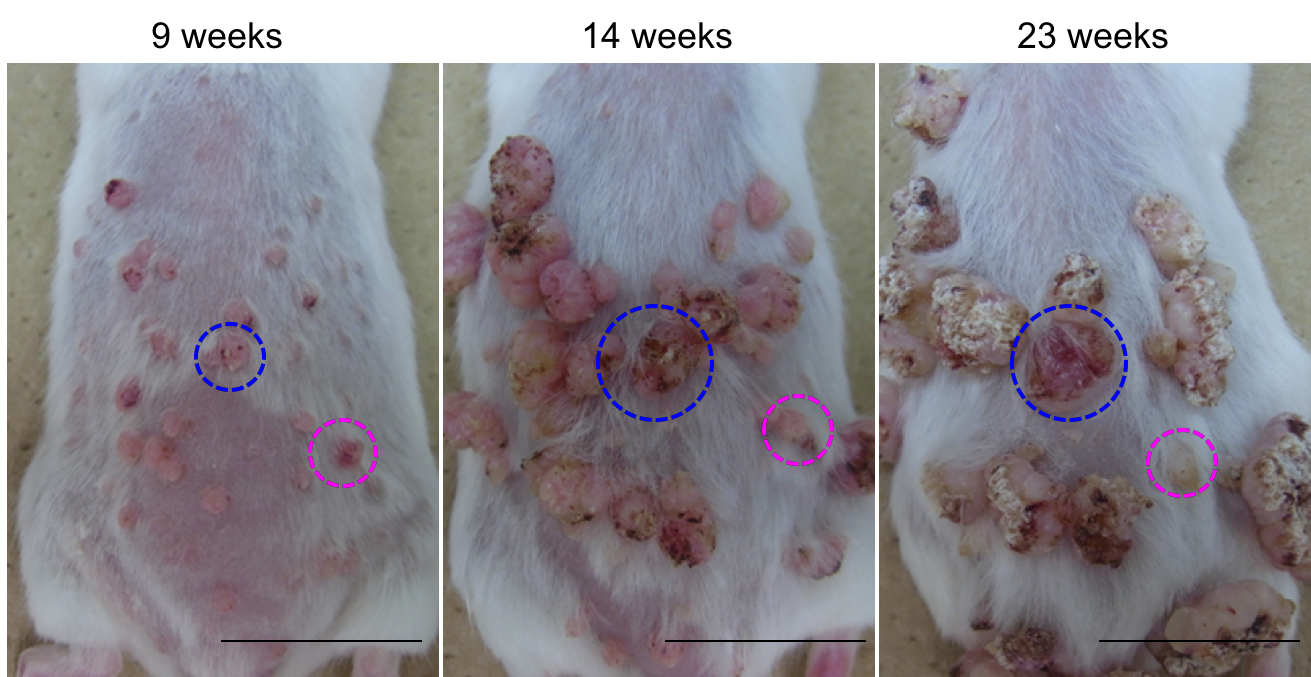


**Supplementary Figure S1.** Photographic images of papillomas and carcinoma in the two-stage skin carcinogenesis by DMBA-TPA protocol. Papillomas appear as outgrowths on the dorsal back skin of mouse. The blue circles indicate malignant series tumor. The pink circles indicate regressed series tumors. Scale bar = 20 mm

**Supplementary Figure S2.** Substitution patterns of SNVs. The detected SNVs were categorized by the substitution patterns. The bar-plot denotes the count of each substitution pattern for each tumor sample.

**Supplementary Figure S3.** Scatter-plot of normalized VAFs of 83 mutation candidate positions. The normalized VAFs of the 83 mutation candidate positions were plotted. Each dot corresponds to a certain genomic position so that 83 plots are drawn in each of the boxes. If the VAFs of a certain position was same value between tumors, the corresponded dot should be on the diagonal line.

**Supplementary Tables**

**Supplementary Table S1.** List of target genes.

| ENSEMBL GENE ID | GENE SYMBOL | ENSEMBL GENE ID | GENE SYMBOL |
| --- | --- | --- | --- |
| ENSMUSG00000000088 | Cox5a | ENSMUSG00000028465 | Tln1 |
| ENSMUSG00000000326 | Comt | ENSMUSG00000028478 | Clta |
| ENSMUSG00000000346 | Dazap2 | ENSMUSG00000028530 | Jak1 |
| ENSMUSG00000000530 | Acvrl1 | ENSMUSG00000028648 | Ndufs5 |
| ENSMUSG00000000568 | Hnrnpd | ENSMUSG00000028670 | Lypla2 |
| ENSMUSG00000000581 | C1d | ENSMUSG00000028691 | Prdx1 |
| ENSMUSG00000000594 | Gm2a | ENSMUSG00000028692 | Akr1a1 |
| ENSMUSG00000001016 | Ilf2 | ENSMUSG00000028745 | Capzb |
| ENSMUSG00000001082 | Mfsd10 | ENSMUSG00000028755 | Cda |
| ENSMUSG00000001119 | Col6a1 | ENSMUSG00000028757 | Ddost |
| ENSMUSG00000001127 | Araf | ENSMUSG00000028798 | Eif3i |
| ENSMUSG00000001150 | Mcm3ap | ENSMUSG00000028809 | Srrm1 |
| ENSMUSG00000001270 | Ckb | ENSMUSG00000028811 | Yars |
| ENSMUSG00000001289 | Pfdn5 | ENSMUSG00000028837 | Psmb2 |
| ENSMUSG00000001416 | Cct3 | ENSMUSG00000028893 | Sesn2 |
| ENSMUSG00000001472 | Tcf25 | ENSMUSG00000028955 | Vamp3 |
| ENSMUSG00000001517 | Foxm1 | ENSMUSG00000028964 | Park7 |
| ENSMUSG00000001761 | Smo | ENSMUSG00000028980 | H6pd |
| ENSMUSG00000001786 | Fbxo7 | ENSMUSG00000028991 | Mtor |
| ENSMUSG00000001833 | Sept7 | ENSMUSG00000028998 | Tomm7 |
| ENSMUSG00000001847 | Rac1 | ENSMUSG00000029020 | Mfn2 |
| ENSMUSG00000001870 | Ltbp1 | ENSMUSG00000029062 | Cdk11b |
| ENSMUSG00000002015 | Bcap31 | ENSMUSG00000029070 | Mxra8 |
| ENSMUSG00000002413 | Braf | ENSMUSG00000029106 | Add1 |
| ENSMUSG00000002504 | Slc9a3r2 | ENSMUSG00000029131 | Dnajb6 |
| ENSMUSG00000002524 | Puf60 | ENSMUSG00000029231 | Pdgfra |
| ENSMUSG00000002768 | Mea1 | ENSMUSG00000029465 | Arpc3 |
| ENSMUSG00000002778 | Kdelr1 | ENSMUSG00000029472 | Anapc5 |
| ENSMUSG00000002814 | Top3a | ENSMUSG00000029538 | Srsf9 |
| ENSMUSG00000002949 | Timm44 | ENSMUSG00000029580 | Actb |
| ENSMUSG00000003037 | Rab8a | ENSMUSG00000029616 | Erp29 |
| ENSMUSG00000003068 | Stk11 | ENSMUSG00000029623 | Pdap1 |
| ENSMUSG00000003072 | Atp5d | ENSMUSG00000029713 | Gnb2 |
| ENSMUSG00000003344 | Btbd2 | ENSMUSG00000030007 | Cct7 |
| ENSMUSG00000003378 | Grik5 | ENSMUSG00000030120 | Mlf2 |
| ENSMUSG00000003380 | Rabac1 | ENSMUSG00000030180 | Kdm5a |
| ENSMUSG00000003402 | Prkcsh | ENSMUSG00000030201 | Lrp6 |
| ENSMUSG00000003410 | Elavl3 | ENSMUSG00000030246 | Ldhb |
| ENSMUSG00000003528 | Slc25a1 | ENSMUSG00000030265 | Kras |
| ENSMUSG00000003813 | Rad23a | ENSMUSG00000030337 | Vamp1 |
| ENSMUSG00000003868 | Ruvbl2 | ENSMUSG00000030591 | Psmd8 |
| ENSMUSG00000003970 | Rpl8 | ENSMUSG00000030602 | Pak4 |
| ENSMUSG00000004054 | Map3k11 | ENSMUSG00000030678 | Maz |
| ENSMUSG00000004264 | Phb2 | ENSMUSG00000030795 | Fus |
| ENSMUSG00000004937 | Sgta | ENSMUSG00000030849 | Fgfr2 |
| ENSMUSG00000005043 | Sgsh | ENSMUSG00000030890 | Ilk |
| ENSMUSG00000005054 | Cstb | ENSMUSG00000030954 | Gp2 |
| ENSMUSG00000005103 | Wdr1 | ENSMUSG00000031024 | St5 |
| ENSMUSG00000005198 | Polr2a | ENSMUSG00000031029 | Eif3f |
| ENSMUSG00000005370 | Msh6 | ENSMUSG00000031066 | Usp11 |
| ENSMUSG00000005483 | Dnajb1 | ENSMUSG00000031167 | Rbm3 |
| ENSMUSG00000005540 | Fcer2a | ENSMUSG00000031207 | Msn |
| ENSMUSG00000005566 | Trim28 | ENSMUSG00000031311 | Nono |
| ENSMUSG00000005575 | Ube2m | ENSMUSG00000031447 | Lamp1 |
| ENSMUSG00000005610 | Eif4g2 | ENSMUSG00000031511 | Arhgef7 |
| ENSMUSG00000005621 | Zfp592 | ENSMUSG00000031535 | Dkk4 |
| ENSMUSG00000005672 | Kit | ENSMUSG00000031586 | Rbpms |
| ENSMUSG00000005779 | Psmb4 | ENSMUSG00000031672 | Got2 |
| ENSMUSG00000005871 | Apc | ENSMUSG00000031729 | Ist1 |
| ENSMUSG00000006057 | Atp5g1 | ENSMUSG00000031760 | Mt3 |
| ENSMUSG00000006095 | Tbcb | ENSMUSG00000031776 | Arl2bp |
| ENSMUSG00000006304 | Arpc2 | ENSMUSG00000031785 | Gpr56 |
| ENSMUSG00000006315 | Tmem147 | ENSMUSG00000031788 | Kifc3 |
| ENSMUSG00000006333 | Rps9 | ENSMUSG00000031818 | Cox4i1 |
| ENSMUSG00000006344 | Ggt5 | ENSMUSG00000031839 | Hsbp1 |
| ENSMUSG00000006498 | Ptbp1 | ENSMUSG00000031948 | Kars |
| ENSMUSG00000006932 | Ctnnb1 | ENSMUSG00000031950 | Gabarapl2 |
| ENSMUSG00000007458 | M6pr | ENSMUSG00000031990 | Jam3 |
| ENSMUSG00000007564 | Ppp2r1a | ENSMUSG00000031996 | Aplp2 |
| ENSMUSG00000007850 | Hnrnph1 | ENSMUSG00000032085 | Tagln |
| ENSMUSG00000007872 | Id3 | ENSMUSG00000032115 | Hyou1 |
| ENSMUSG00000007891 | Ctsd | ENSMUSG00000032231 | Anxa2 |
| ENSMUSG00000008036 | Ap2s1 | ENSMUSG00000032294 | Pkm |
| ENSMUSG00000008958 | Vps72 | ENSMUSG00000032312 | Csk |
| ENSMUSG00000009073 | Nf2 | ENSMUSG00000032330 | Cox7a2 |
| ENSMUSG00000009090 | Ap1b1 | ENSMUSG00000032356 | Rasgrf1 |
| ENSMUSG00000009291 | Pttg1ip | ENSMUSG00000032423 | Syncrip |
| ENSMUSG00000009470 | Tnpo1 | ENSMUSG00000032479 | Mtap4 |
| ENSMUSG00000009549 | Srp14 | ENSMUSG00000032480 | Dhx30 |
| ENSMUSG00000009630 | Ppp2cb | ENSMUSG00000032498 | Mlh1 |
| ENSMUSG00000009927 | Rps25 | ENSMUSG00000032562 | Gnai2 |
| ENSMUSG00000010097 | Nxf1 | ENSMUSG00000032637 | Atxn2l |
| ENSMUSG00000010376 | Nedd8 | ENSMUSG00000032737 | Inppl1 |
| ENSMUSG00000012405 | Rpl15 | ENSMUSG00000032845 | Alpk2 |
| ENSMUSG00000012535 | Tnpo3 | ENSMUSG00000033020 | Polr2f |
| ENSMUSG00000012848 | Rps5 | ENSMUSG00000033068 | Entpd6 |
| ENSMUSG00000013663 | Pten | ENSMUSG00000033159 | Cnppd1 |
| ENSMUSG00000014294 | Ndufa2 | ENSMUSG00000033379 | Atp6v0b |
| ENSMUSG00000014426 | Map3k4 | ENSMUSG00000033430 | Terf2ip |
| ENSMUSG00000014599 | Csf1 | ENSMUSG00000033569 | Bai3 |
| ENSMUSG00000014606 | Slc25a11 | ENSMUSG00000033809 | Alg3 |
| ENSMUSG00000014748 | Tex261 | ENSMUSG00000033826 | Dnahc8 |
| ENSMUSG00000014769 | Psmb1 | ENSMUSG00000033916 | Chmp2a |
| ENSMUSG00000014859 | E2f4 | ENSMUSG00000033938 | Ndufb7 |
| ENSMUSG00000015120 | Ube2i | ENSMUSG00000034165 | Ccnd3 |
| ENSMUSG00000015291 | Gdi1 | ENSMUSG00000034210 | 4732418C07Rik |
| ENSMUSG00000015605 | Srf | ENSMUSG00000034659 | Tmem109 |
| ENSMUSG00000015721 | Nlrp5 | ENSMUSG00000034681 | Rnps1 |
| ENSMUSG00000015937 | H2afy | ENSMUSG00000034868 | Myl12b |
| ENSMUSG00000016427 | Ndufa1 | ENSMUSG00000034928 | Rnf44 |
| ENSMUSG00000016528 | Mapkapk2 | ENSMUSG00000035027 | Map2k2 |
| ENSMUSG00000016554 | Eif3d | ENSMUSG00000035086 | Becn1 |
| ENSMUSG00000017390 | Aldoc | ENSMUSG00000035112 | Wnk4 |
| ENSMUSG00000017404 | Rpl19 | ENSMUSG00000035354 | Uvrag |
| ENSMUSG00000017428 | Psmd11 | ENSMUSG00000035885 | Cox8a |
| ENSMUSG00000017652 | Cd40 | ENSMUSG00000036111 | Lmo1 |
| ENSMUSG00000018287 | Spag7 | ENSMUSG00000036606 | Plxnb2 |
| ENSMUSG00000018326 | Ywhab | ENSMUSG00000036712 | Cyld |
| ENSMUSG00000018340 | Anxa6 | ENSMUSG00000036721 | Zscan12 |
| ENSMUSG00000018537 | Pcgf2 | ENSMUSG00000036751 | Cox6b1 |
| ENSMUSG00000018559 | Ctdnep1 | ENSMUSG00000037152 | Ndufc1 |
| ENSMUSG00000018567 | Gabarap | ENSMUSG00000037206 | Islr |
| ENSMUSG00000018697 | Aatf | ENSMUSG00000037337 | Map4k1 |
| ENSMUSG00000018770 | Atp5g3 | ENSMUSG00000037362 | Nov |
| ENSMUSG00000018965 | Ywhah | ENSMUSG00000037373 | Ctbp1 |
| ENSMUSG00000019087 | Atp6ap1 | ENSMUSG00000037563 | Rps16 |
| ENSMUSG00000019210 | Atp6v1e1 | ENSMUSG00000037646 | Vps13b |
| ENSMUSG00000019461 | Plscr3 | ENSMUSG00000037706 | Cd81 |
| ENSMUSG00000019494 | Cops6 | ENSMUSG00000037788 | Vopp1 |
| ENSMUSG00000019804 | Snx3 | ENSMUSG00000037916 | Ndufv1 |
| ENSMUSG00000019889 | Ptprk | ENSMUSG00000038346 | Zfp384 |
| ENSMUSG00000020122 | Egfr | ENSMUSG00000038366 | Lasp1 |
| ENSMUSG00000020149 | Rab1 | ENSMUSG00000038462 | Uqcrfs1 |
| ENSMUSG00000020163 | Uqcr11 | ENSMUSG00000038489 | Polr2l |
| ENSMUSG00000020267 | Hint1 | ENSMUSG00000038502 | Ptov1 |
| ENSMUSG00000020287 | Mpg | ENSMUSG00000038612 | Mcl1 |
| ENSMUSG00000020321 | Mdh1 | ENSMUSG00000038618 | Rassf7 |
| ENSMUSG00000020358 | Hnrnpab | ENSMUSG00000038619 | Ensa |
| ENSMUSG00000020368 | Canx | ENSMUSG00000038650 | Rnh1 |
| ENSMUSG00000020372 | Gnb2l1 | ENSMUSG00000038690 | Atp5j2 |
| ENSMUSG00000020395 | Itk | ENSMUSG00000038909 | Myst2 |
| ENSMUSG00000020440 | Arf5 | ENSMUSG00000038970 | Lmtk2 |
| ENSMUSG00000020444 | Guk1 | ENSMUSG00000039105 | Atp6v1g1 |
| ENSMUSG00000020458 | Rtn4 | ENSMUSG00000039195 | 1110008P14Rik |
| ENSMUSG00000020484 | Xbp1 | ENSMUSG00000039205 | Ciz1 |
| ENSMUSG00000020538 | Srebf1 | ENSMUSG00000039262 | Prrc2b |
| ENSMUSG00000020580 | Rock2 | ENSMUSG00000039452 | Snx22 |
| ENSMUSG00000020673 | Tpo | ENSMUSG00000039488 | Cntn5 |
| ENSMUSG00000020695 | Mrc2 | ENSMUSG00000039831 | Arhgap29 |
| ENSMUSG00000020716 | Nf1 | ENSMUSG00000039852 | Rere |
| ENSMUSG00000020738 | Sumo2 | ENSMUSG00000039953 | Clstn1 |
| ENSMUSG00000020821 | Kif1c | ENSMUSG00000040158 | Tax1bp3 |
| ENSMUSG00000020850 | Prpf8 | ENSMUSG00000040488 | Ltbp4 |
| ENSMUSG00000021025 | Nfkbia | ENSMUSG00000040521 | Tsfm |
| ENSMUSG00000021037 | Ahsa1 | ENSMUSG00000040537 | Adam22 |
| ENSMUSG00000021127 | Zfp36l1 | ENSMUSG00000040614 | Nlrp9c |
| ENSMUSG00000021144 | Mta1 | ENSMUSG00000040687 | Madd |
| ENSMUSG00000021218 | Gdi2 | ENSMUSG00000040836 | Gpr161 |
| ENSMUSG00000021376 | Tpmt | ENSMUSG00000041075 | Fzd7 |
| ENSMUSG00000021466 | Ptch1 | ENSMUSG00000041126 | H2afv |
| ENSMUSG00000021518 | Ptdss1 | ENSMUSG00000041237 | Pklr |
| ENSMUSG00000021576 | Pdcd6 | ENSMUSG00000041278 | Ttc1 |
| ENSMUSG00000021577 | Sdha | ENSMUSG00000041355 | Ssr2 |
| ENSMUSG00000021614 | Vcan | ENSMUSG00000041596 | Vmn1r90 |
| ENSMUSG00000021877 | Arf4 | ENSMUSG00000041881 | Ndufa7 |
| ENSMUSG00000021978 | Extl3 | ENSMUSG00000041939 | Mvk |
| ENSMUSG00000022100 | Xpo7 | ENSMUSG00000042406 | Atf4 |
| ENSMUSG00000022105 | Rb1 | ENSMUSG00000043079 | Synpo |
| ENSMUSG00000022174 | Dad1 | ENSMUSG00000045284 | Dcaf12l1 |
| ENSMUSG00000022194 | Pabpn1 | ENSMUSG00000045569 | Mc2r |
| ENSMUSG00000022212 | Cpne6 | ENSMUSG00000046449 | C77370 |
| ENSMUSG00000022223 | Sdr39u1 | ENSMUSG00000046865 | Fbl |
| ENSMUSG00000022285 | Ywhaz | ENSMUSG00000046982 | Tshz1 |
| ENSMUSG00000022311 | Csmd3 | ENSMUSG00000047547 | Cltb |
| ENSMUSG00000022433 | Csnk1e | ENSMUSG00000047649 | Cd3eap |
| ENSMUSG00000022443 | Myh9 | ENSMUSG00000047945 | Marcksl1 |
| ENSMUSG00000022471 | Xrcc6 | ENSMUSG00000048076 | Arf1 |
| ENSMUSG00000022521 | Crebbp | ENSMUSG00000048154 | Mll2 |
| ENSMUSG00000022561 | Gpaa1 | ENSMUSG00000048578 | Mlec |
| ENSMUSG00000022570 | Tsta3 | ENSMUSG00000048930 | Tada3 |
| ENSMUSG00000022672 | Prkdc | ENSMUSG00000048960 | Prex2 |
| ENSMUSG00000022841 | Ap2m1 | ENSMUSG00000050310 | Rictor |
| ENSMUSG00000022884 | Eif4a2 | ENSMUSG00000050824 | Sstr5 |
| ENSMUSG00000022982 | Sod1 | ENSMUSG00000050856 | Atp5k |
| ENSMUSG00000023010 | Tmbim6 | ENSMUSG00000050926 | Dcaf12l2 |
| ENSMUSG00000023175 | Bsg | ENSMUSG00000051306 | Usp42 |
| ENSMUSG00000023903 | Mmp25 | ENSMUSG00000051747 | Ttn |
| ENSMUSG00000023944 | Hsp90ab1 | ENSMUSG00000051853 | Arf3 |
| ENSMUSG00000024073 | Birc6 | ENSMUSG00000052026 | Slc6a7 |
| ENSMUSG00000024121 | Atp6v0c | ENSMUSG00000052397 | Ezr |
| ENSMUSG00000024213 | Nudt3 | ENSMUSG00000052423 | B4galt3 |
| ENSMUSG00000024248 | Cox7a2l | ENSMUSG00000052429 | Prmt1 |
| ENSMUSG00000024346 | Pfdn1 | ENSMUSG00000052681 | Rap1b |
| ENSMUSG00000024456 | Diap1 | ENSMUSG00000052713 | Zfp608 |
| ENSMUSG00000024483 | Ankhd1 | ENSMUSG00000052957 | Gas1 |
| ENSMUSG00000024525 | Impa2 | ENSMUSG00000053141 | Ptprt |
| ENSMUSG00000024527 | Afg3l2 | ENSMUSG00000053399 | Adamts18 |
| ENSMUSG00000024613 | Tcof1 | ENSMUSG00000053560 | Ier2 |
| ENSMUSG00000024639 | Gnaq | ENSMUSG00000053565 | Eif3k |
| ENSMUSG00000024646 | Cyb5 | ENSMUSG00000054102 | Nlrp9a |
| ENSMUSG00000024767 | Otub1 | ENSMUSG00000054252 | Fgfr3 |
| ENSMUSG00000024792 | Zfpl1 | ENSMUSG00000054452 | Aes |
| ENSMUSG00000024824 | Rad9 | ENSMUSG00000055022 | Cntn1 |
| ENSMUSG00000024826 | Dpf2 | ENSMUSG00000055681 | Cope |
| ENSMUSG00000024830 | Rps6kb2 | ENSMUSG00000057177 | Gsk3a |
| ENSMUSG00000024851 | Pitpnm1 | ENSMUSG00000057278 | Snrpg |
| ENSMUSG00000024858 | Adrbk1 | ENSMUSG00000058454 | Dhcr7 |
| ENSMUSG00000024914 | Drap1 | ENSMUSG00000058558 | Rpl5 |
| ENSMUSG00000024927 | Rela | ENSMUSG00000058569 | Tmed9 |
| ENSMUSG00000024944 | Arl2 | ENSMUSG00000058655 | Eif4b |
| ENSMUSG00000024962 | Vegfb | ENSMUSG00000059070 | Rpl18 |
| ENSMUSG00000025130 | P4hb | ENSMUSG00000059447 | Hadhb |
| ENSMUSG00000025132 | Arhgdia | ENSMUSG00000059518 | Znhit1 |
| ENSMUSG00000025231 | Sufu | ENSMUSG00000059552 | Trp53 |
| ENSMUSG00000025321 | Itgb8 | ENSMUSG00000059708 | Akap17b |
| ENSMUSG00000025366 | Esyt1 | ENSMUSG00000060429 | Sntb1 |
| ENSMUSG00000025381 | Cnpy2 | ENSMUSG00000060508 | Nlrp9b |
| ENSMUSG00000025428 | Atp5a1 | ENSMUSG00000060680 | Gm8894 |
| ENSMUSG00000025474 | Tubgcp2 | ENSMUSG00000060802 | B2m |
| ENSMUSG00000025499 | Hras1 | ENSMUSG00000061315 | Naca |
| ENSMUSG00000025503 | Taldo1 | ENSMUSG00000061462 | Obscn |
| ENSMUSG00000025508 | Rplp2 | ENSMUSG00000061904 | Slc25a3 |
| ENSMUSG00000025651 | Uqcrc1 | ENSMUSG00000062070 | Pgk1 |
| ENSMUSG00000025743 | Sdc3 | ENSMUSG00000062284 | Gm6030 |
| ENSMUSG00000025745 | Hadha | ENSMUSG00000062825 | Actg1 |
| ENSMUSG00000025793 | Hgs | ENSMUSG00000062867 | Impdh2 |
| ENSMUSG00000025892 | Gria4 | ENSMUSG00000063229 | Ldha |
| ENSMUSG00000026024 | Als2 | ENSMUSG00000063239 | Grm4 |
| ENSMUSG00000026209 | Dnpep | ENSMUSG00000063410 | Stk24 |
| ENSMUSG00000026276 | Sept2 | ENSMUSG00000063457 | Rps15 |
| ENSMUSG00000026395 | Ptprc | ENSMUSG00000063511 | Snrnp70 |
| ENSMUSG00000026450 | Chit1 | ENSMUSG00000063870 | Chd4 |
| ENSMUSG00000026750 | Psmb7 | ENSMUSG00000063882 | Uqcrh |
| ENSMUSG00000026786 | Apbb1ip | ENSMUSG00000064068 | Mtx1 |
| ENSMUSG00000026842 | Abl1 | ENSMUSG00000065954 | Tacc1 |
| ENSMUSG00000026864 | Hspa5 | ENSMUSG00000066306 | Numa1 |
| ENSMUSG00000026976 | Pax8 | ENSMUSG00000067150 | Xpo5 |
| ENSMUSG00000027193 | Api5 | ENSMUSG00000067657 | AC124479.2 |
| ENSMUSG00000027223 | Mapk8ip1 | ENSMUSG00000067713 | Prkag1 |
| ENSMUSG00000027224 | Duoxa1 | ENSMUSG00000068206 | Pick1 |
| ENSMUSG00000027247 | Arhgap1 | ENSMUSG00000068267 | Cenpb |
| ENSMUSG00000027276 | Jag1 | ENSMUSG00000070319 | Eif3g |
| ENSMUSG00000027367 | Stard7 | ENSMUSG00000071054 | Safb |
| ENSMUSG00000027404 | Snrpb | ENSMUSG00000071076 | Jund |
| ENSMUSG00000027406 | Idh3b | ENSMUSG00000071531 | Gprin2 |
| ENSMUSG00000027422 | Rrbp1 | ENSMUSG00000071645 | Tut1 |
| ENSMUSG00000027523 | Gnas | ENSMUSG00000071650 | Ganab |
| ENSMUSG00000027566 | Psma7 | ENSMUSG00000073226 | Gm10482 |
| ENSMUSG00000027665 | Pik3ca | ENSMUSG00000073640 | Rpl27-ps3 |
| ENSMUSG00000027852 | Nras | ENSMUSG00000073802 | Cdkn2b |
| ENSMUSG00000027937 | Jtb | ENSMUSG00000074034 | Gm5921 |
| ENSMUSG00000027944 | Hax1 | ENSMUSG00000074305 | C230081A13Rik |
| ENSMUSG00000027951 | Adar | ENSMUSG00000074643 | Cpne1 |
| ENSMUSG00000028039 | Efna3 | ENSMUSG00000075706 | Gpx4 |
| ENSMUSG00000028041 | Adam15 | ENSMUSG00000076432 | Ywhaq |
| ENSMUSG00000028049 | Scamp3 | ENSMUSG00000078676 | Casc3 |
| ENSMUSG00000028140 | Mrpl9 | ENSMUSG00000079426 | Arpc4 |
| ENSMUSG00000028289 | Epha7 | ENSMUSG00000079523 | Tmsb10 |
| ENSMUSG00000028312 | Smc2 | ENSMUSG00000089986 | Ankhd1-Eif4ebp3 |
| ENSMUSG00000028367 | Txn1 | ENSMUSG00000090247 | Bloc1s1 |

We designed the custom bait library by using Agilent SureDesign. The whole gene regions of these 500 genes, including the both exon and intron, were targeted. The genes which listed in the COSMIC: Cancer Gene Census were highlighted in red.

**Supplementary Table S2.** Sequencing statistics for each sample.

| Sample name | # clusters | # reads | # QC | %QC | %duplicates |
| --- | --- | --- | --- | --- | --- |
| Papi9w | 29,494,118 | 58,988,236 | 35,567,485 | 60.30 | 33.99 |
| Papi14w | 26,126,652 | 52,253,304 | 33,379,356 | 63.88 | 28.13 |
| Carc | 28,909,899 | 57,819,798 | 39,688,768 | 68.64 | 23.08 |
| Meta | 31,889,353 | 63,778,706 | 36,805,260 | 57.71 | 34.39 |
| Reg9w | 40,090,049 | 80,180,098 | 51,276,309 | 63.95 | 24.28 |
| Reg14w | 31,473,369 | 62,946,738 | 32,821,844 | 52.14 | 37.64 |
| Tail (ctrl) | 31,218,916 | 62,437,832 | 30,439,480 | 48.75 | 45.13 |
| Ave. | 31,314,622 |  |  | 59.34 | 32.38 |

The sample names correspond to those of Fig 1a. The column # QC (quality control) denotes the number of reads that remained after the pre-processing to exclude low-quality sequenced reads. The column %QC denotes the percentage of reads remaining after the pre-processing. The column “%duplicates” denotes the percentage of the duplicated reads for each sample, which was calculated from the number of filtered reads in the de-duplicate process.

**Supplementary Table S3.** Sequencing depth of 83 mutation candidate position for each sample.

| **Sample name** | **Max** | **Min** | **Average** | **Median** |
| --- | --- | --- | --- | --- |
| Papi9w | 976 | 52 | 349.76 | 265 |
| Papi14w | 2152 | 52 | 461.87 | 332 |
| Carc | 1093 | 72 | 420.64 | 319 |
| Meta | 1133 | 65 | 438.46 | 309 |
| Reg9w | 2165 | 55 | 543.08 | 459 |
| Reg14w | 2654 | 58 | 527.72 | 368 |
| Tail (ctrl) | 1236 | 55 | 407.33 | 260 |

The statistics of the coverage of the 83 mutation candidate positions for each sample are shown.

**Supplementary Table S4.** Tumor purities and normalization factors.

| **Sample name** | **# Mapped reads** | **# Variant reads** | **Raw VAF (%)** | **95% confidence interval (%)** | **Tumor purity (%)** | **NF** |
| --- | --- | --- | --- | --- | --- | --- |
| Papi9w | 912 | 342 | 37.500 | 34.35 – 40.73 | 68.70 – 81.47 | 1.3333 |
| Papi14w | 2152 | 974 | 45.260 | 43.14 – 47.39 | 86.28 – 94.78 | 1.1047 |
| Carc | 910 | 277 | 30.440 | 27.46 – 33.54 | 54.92 – 67.09 | 1.6426 |
| Meta | 836 | 62 | 7.4163 | 5.733 – 9.411 | 11.47 – 18.81 | 6.7419 |
| Reg9w | 1271 | 648 | 50.983 | 48.20 – 53.77 | 96.39 – 100.0 | 0.9807 |
| Reg14w | 1553 | 522 | 33.612 | 31.26 – 36.02 | 62.53 – 72.05 | 1.4875 |

The number of mapped reads on the Q61L *Hras* mutation position (chr7:141192550), the number of variant reads which support the *Hras* mutation, the raw VAF of the *Hras* mutation, the 95% confidence interval of the VAF, estimated tumor purity, and normalization factor (NF) for each tumor sample are shown in this table. The heterozygous *Hras* mutation is an initiator of the experimental carcinogenesis protocol we used; therefore, the VAF of the *Hras* mutation should be 50%. Then, we calculated the normalization factor by 0.5 / (raw VAF). We used up to the seventh decimal place of the NFs to minimize the effect of rounding errors when we calculated the normalized VAFs.

**Supplementary Table S5.** Details of 83 mutation candidate positions.

| position | Normalized VAF | | | | | | Annotation | |
| --- | --- | --- | --- | --- | --- | --- | --- | --- |
|  | Papi 9w | Papi 14w | Carc | Meta | Reg 9w | Reg 14w | Effect | GeneID |
| 7:141192550 | 0.4999 | 0.5001 | 0.5001 | 0.5000 | 0.5001 | 0.5001 | Missense | Hras1 |
| 2:98662257 | 0.0259 | 0.0197 | 0.0611 | 0.0000 | 0.0224 | 0.0268 | Synonymous | Gm10801 |
| 2:98662457 | 0.0396 | 0.0000 | 0.0442 | 0.1711 | 0.0205 | 0.0453 | Missense | Gm10801 |
| 9:3000377 | 0.0368 | 0.0403 | 0.0330 | 0.2766 | 0.0289 | 0.0362 | Upstream | Gm10722 |
| 2:98662399 | 0.0302 | 0.0197 | 0.0696 | 0.1822 | 0.0274 | 0.0472 | Missense | Gm10801 |
| 9:35305329 | 0.0222 | 0.0108 | 0.0752 | 0.2000 | 0.0204 | 0.0420 | Intergenic | - |
| 9:35305343 | 0.0237 | 0.0254 | 0.0342 | 0.0862 | 0.0154 | 0.0257 | Intergenic | - |
| 2:98665129 | 0.0284 | 0.0220 | 0.0451 | 0.1162 | 0.0191 | 0.0432 | Downstream | Gm10800 |
| X:163478989 | 0.0369 | 0.0205 | 0.0568 | 0.0992 | 0.0138 | 0.0421 | Others | Gm7199 |
| 2:98662293 | 0.0199 | 0.0090 | 0.0360 | 0.1926 | 0.0138 | 0.0183 | Synonymous | Gm10801 |
| 2:98666287 | 0.0213 | 0.0071 | 0.0483 | 0.0940 | 0.0269 | 0.0282 | Downstream | Gm10800 |
| 2:98662411 | 0.0000 | 0.0126 | 0.0592 | 0.1174 | 0.0186 | 0.0257 | Missense | Gm10801 |
| 2:98662805 | 0.0000 | 0.0505 | 0.0348 | 0.2408 | 0.0271 | 0.0765 | Downstream | Gm10800 |
| 14:19417883 | 0.0441 | 0.0000 | 0.0000 | 0.2345 | 0.0152 | 0.0213 | Intron | AC242409.1 |
| 9:3025531 | 0.0000 | 0.0000 | 0.0000 | 0.2437 | 0.0277 | 0.0461 | Missense | Gm10716 |
| 1:181187046 | 0.0000 | 0.0390 | 0.0000 | 0.1644 | 0.0164 | 0.0000 | Upstream | Wdr26 |
| 9:3002159 | 0.0000 | 0.0391 | 0.0692 | 0.0000 | 0.0177 | 0.0198 | Upstream | Gm11168 |
| 9:3002107 | 0.0000 | 0.0567 | 0.0000 | 0.0000 | 0.0149 | 0.0188 | Upstream | Gm11168 |
| 9:3002141 | 0.0000 | 0.0337 | 0.0000 | 0.0000 | 0.0206 | 0.0211 | Upstream | Gm11168 |
| 14:65077037 | 0.0130 | 0.0040 | 0.0000 | 0.0179 | 0.0042 | 0.0000 | Missense | Extl3 |
| 19:8766798 | 0.0109 | 0.0000 | 0.0000 | 0.0259 | 0.0036 | 0.0000 | Missense | Nxf1 |
| 18:15414888 | 0.0000 | 0.3858 | 0.1519 | 0.0000 | 0.0000 | 0.0000 | Intergenic | - |
| 5:10957983 | 0.0000 | 0.2231 | 0.0865 | 0.0000 | 0.0000 | 0.0000 | Intron | Gm5152 |
| 1:138067914 | 0.0000 | 0.3376 | 0.2823 | 0.3540 | 0.0000 | 0.0000 | Nonsense | Ptprc |
| 15:79144413 | 0.0000 | 0.3357 | 0.3370 | 0.3548 | 0.0000 | 0.0000 | Intron | Polr2f |
| 2:76896947 | 0.0000 | 0.3243 | 0.1524 | 0.1359 | 0.0000 | 0.0000 | Missense | Ttn |
| 5:84866414 | 0.0000 | 0.2879 | 0.1527 | 0.1341 | 0.0000 | 0.0000 | Intergenic | - |
| 14:69159185 | 0.0000 | 0.2684 | 0.1377 | 0.1117 | 0.0000 | 0.0000 | Intergenic | - |
| 7:39662651 | 0.0000 | 0.2042 | 0.1498 | 0.1171 | 0.0000 | 0.0000 | Intergenic | - |
| 12:30092627 | 0.0000 | 0.2944 | 0.1371 | 0.1996 | 0.0000 | 0.0000 | Synonymous | Tpo |
| 8:88710071 | 0.0000 | 0.3413 | 0.1442 | 0.1922 | 0.0000 | 0.0000 | Intron | Cyld |
| 10:29781362 | 0.0000 | 0.4175 | 0.1781 | 0.1993 | 0.0000 | 0.0000 | Intergenic | - |
| 19:5647309 | 0.0000 | 0.3744 | 0.1837 | 0.2367 | 0.0000 | 0.0000 | Missense | Rela |
| 4:43554186 | 0.0000 | 0.3647 | 0.2011 | 0.1795 | 0.0000 | 0.0000 | Upstream | Tln1 |
| 9:88454294 | 0.0000 | 0.3259 | 0.2016 | 0.1928 | 0.0000 | 0.0000 | Intron | Gm20537 |
| 2:76807228 | 0.0000 | 0.3395 | 0.1745 | 0.1402 | 0.0000 | 0.0000 | Intron | Ttn |
| 2:91178303 | 0.0000 | 0.3282 | 0.1876 | 0.1712 | 0.0000 | 0.0000 | Missense | Madd |
| 4:28817318 | 0.0000 | 0.3509 | 0.1860 | 0.1568 | 0.0000 | 0.0000 | Intron | Epha7 |
| 15:35705682 | 0.0000 | 0.0000 | 0.0213 | 0.0000 | 0.0000 | 0.0000 | Intron | Vps13b |
| 17:12247878 | 0.0000 | 0.0000 | 0.0755 | 0.0000 | 0.0000 | 0.0000 | Intron | Map3k4 |
| 8:43289942 | 0.0000 | 0.0000 | 0.0438 | 0.0000 | 0.0000 | 0.0000 | Intergenic | - |
| 15:89160554 | 0.0000 | 0.0000 | 0.0000 | 0.1282 | 0.0000 | 0.0000 | Upstream | Plxnb2 |
| 11:120346973 | 0.0000 | 0.0000 | 0.0405 | 0.1253 | 0.0000 | 0.0000 | Missense | Actg1 |
| 11:120346787 | 0.0000 | 0.0000 | 0.0123 | 0.0570 | 0.0000 | 0.0000 | Synonymous | Actg1 |
| 8:100030643 | 0.0000 | 0.0000 | 0.0153 | 0.0672 | 0.0000 | 0.0000 | Others | Gm15210 |
| 2:76787076 | 0.0000 | 0.0000 | 0.0000 | 0.0000 | 0.3917 | 0.4303 | Synonymous | Ttn |
| 6:125218819 | 0.0000 | 0.0000 | 0.0000 | 0.0000 | 0.3908 | 0.3274 | Intron | Vamp1 |
| 12:18013571 | 0.0000 | 0.0000 | 0.0000 | 0.0000 | 0.3883 | 0.3083 | Intergenic | - |
| 15:98864350 | 0.0000 | 0.0000 | 0.0000 | 0.0000 | 0.3936 | 0.3162 | Synonymous | Mll2 |
| 16:15833961 | 0.0000 | 0.0000 | 0.0000 | 0.0000 | 0.4440 | 0.3818 | Intron | Prkdc |
| 2:76738847 | 0.0000 | 0.0000 | 0.0000 | 0.0000 | 0.4521 | 0.3405 | Missense | Ttn |
| 9:62842036 | 0.0000 | 0.0068 | 0.0000 | 0.0000 | 0.0046 | 0.0000 | Upstream | Cln6 |
| 7:17139272 | 0.0000 | 0.0000 | 0.0000 | 0.0000 | 0.0357 | 0.0000 | Intergenic | - |
| 11:55004766 | 0.0000 | 0.0000 | 0.0000 | 0.0000 | 0.0083 | 0.0000 | Intron | Anxa6 |
| 11:69588515 | 0.0000 | 0.0000 | 0.0000 | 0.0000 | 0.0127 | 0.0000 | Missense | Trp53 |
| 13:98853314 | 0.1033 | 0.0425 | 0.0000 | 0.0000 | 0.0000 | 0.0513 | Intron | Tnpo1 |
| 2:76766295 | 0.0158 | 0.0062 | 0.0000 | 0.0000 | 0.0000 | 0.0229 | Missense | Ttn |
| 4:148550842 | 0.0208 | 0.0166 | 0.0000 | 0.0000 | 0.0000 | 0.0502 | Downstream/Upstream | Mtor/ U6 |
| 11:79436839 | 0.0000 | 0.0119 | 0.0000 | 0.0000 | 0.0000 | 0.0462 | Intron | Nf1 |
| 12:21394815 | 0.0000 | 0.0572 | 0.0000 | 0.0000 | 0.0000 | 0.1283 | Downstream | Ywhaq |
| 2:108948762 | 0.0324 | 0.0000 | 0.0000 | 0.0000 | 0.0000 | 0.0739 | Upstream | Gm13910 |
| 6:133762795 | 0.0939 | 0.0000 | 0.0000 | 0.0000 | 0.0000 | 0.0770 | Upstream | AC122359.1 |
| 11:59067729 | 0.0000 | 0.0000 | 0.0000 | 0.0000 | 0.0000 | 0.0132 | Missense | Obscn |
| 3:89141498 | 0.0000 | 0.0000 | 0.0000 | 0.0000 | 0.0000 | 0.0131 | Intron | Pklr |
| 4:101161963 | 0.0000 | 0.0000 | 0.0000 | 0.0000 | 0.0000 | 0.0597 | Intron | Jak1 |
| 7:21230248 | 0.0000 | 0.0000 | 0.0000 | 0.0000 | 0.0000 | 0.0687 | Intergenic | - |
| 17:46215562 | 0.0000 | 0.0465 | 0.0000 | 0.0000 | 0.0000 | 0.0000 | Intron | Xpo5 |
| 2:76861409 | 0.0000 | 0.0121 | 0.0000 | 0.0000 | 0.0000 | 0.0000 | Intron | Ttn |
| 7:13030561 | 0.0000 | 0.0064 | 0.0000 | 0.0000 | 0.0000 | 0.0000 | Missense | Trim28 |
| 18:60828610 | 0.0000 | 0.0074 | 0.0000 | 0.0639 | 0.0000 | 0.0000 | Intron | Tcof1 |
| 3:99500989 | 0.0000 | 0.0101 | 0.0000 | 0.0203 | 0.0000 | 0.0000 | Others | M6pr-ps |
| 15:90723932 | 0.2446 | 0.0000 | 0.0000 | 0.0000 | 0.0000 | 0.0000 | Intergenic | - |
| 2:76825807 | 0.4816 | 0.0000 | 0.0000 | 0.0000 | 0.0000 | 0.0000 | Missense | Ttn |
| 10:79710761 | 0.4756 | 0.0000 | 0.0000 | 0.0000 | 0.0000 | 0.0000 | Missense | Bsg |
| 6:107881483 | 0.4772 | 0.0000 | 0.0000 | 0.0000 | 0.0000 | 0.0000 | Intergenic | - |
| 2:76846783 | 0.4504 | 0.0000 | 0.0000 | 0.0000 | 0.0000 | 0.0000 | Synonymous | Ttn |
| 14:55514768 | 0.4244 | 0.0000 | 0.0000 | 0.0000 | 0.0000 | 0.0000 | Downstream | Cpne6/Nrl |
| 4:28966425 | 0.4303 | 0.0000 | 0.0000 | 0.0000 | 0.0000 | 0.0000 | 3'UTR | Epha7 |
| 12:31018401 | 0.0187 | 0.0000 | 0.0000 | 0.0000 | 0.0000 | 0.0000 | Intergenic | - |
| 6:122696935 | 0.0597 | 0.0000 | 0.0000 | 0.0000 | 0.0000 | 0.0000 | Downstream | Gm10420 |
| X:110604447 | 0.1181 | 0.0000 | 0.0000 | 0.0000 | 0.0000 | 0.0000 | Downstream | Gm14886 |
| 10:88247930 | 0.0437 | 0.0484 | 0.0000 | 0.0000 | 0.0000 | 0.0000 | Downstream | Ccdc53 |
| 8:33615918 | 0.0226 | 0.0154 | 0.0000 | 0.0000 | 0.0000 | 0.0000 | Intron | Ppp2cb |

The normalized VAFs of each tumor sample, the effect of the mutation, and the genes including the position are shown. The order corresponds to Fig. 1b.
